# A novel multi-tissue RNA diagnostic of healthy ageing relates to cognitive health status

**DOI:** 10.1186/s13059-015-0750-x

**Published:** 2015-09-07

**Authors:** Sanjana Sood, Iain J. Gallagher, Katie Lunnon, Eric Rullman, Aoife Keohane, Hannah Crossland, Bethan E. Phillips, Tommy Cederholm, Thomas Jensen, Luc JC van Loon, Lars Lannfelt, William E. Kraus, Philip J. Atherton, Robert Howard, Thomas Gustafsson, Angela Hodges, James A. Timmons

**Affiliations:** XRGenomics Ltd, London, UK; Division of Genetics & Molecular Medicine, King’s College London, 8th Floor, Tower Wing, Guy’s Hospital, London, SE1 9RT UK; School of Health, Stirling University, Stirling, Scotland UK; Department of Old Age Psychiatry, King’s College London, London, UK; Division of Clinical Physiology, Karolinska University Hospital, Stockholm, Sweden; School of Medicine, Derby Royal Hospital, Derbyshire, UK; Department of Public Health, Caring Sciences, Clinical Nutrition and Metabolism, Uppsala University, Uppsala, Sweden; Medical Prognosis Institute A/S, Hørsholm, Denmark; NUTRIM, Maastricht University, Maastricht, The Netherlands; Department of Public Health and Caring Sciences/Molecular Geriatrics, Uppsala University, Uppsala, Sweden; Duke Molecular Physiology Institute, Duke University School of Medicine, Durham, NC USA; Present address: University of Exeter Medical School, Exeter, UK

## Abstract

**Background:**

Diagnostics of the human ageing process may help predict future healthcare needs or guide preventative measures for tackling diseases of older age. We take a transcriptomics approach to build the first reproducible multi-tissue RNA expression signature by gene-chip profiling tissue from sedentary normal subjects who reached 65 years of age in good health.

**Results:**

One hundred and fifty probe-sets form an accurate classifier of young versus older muscle tissue and this healthy ageing RNA classifier performed consistently in independent cohorts of human muscle, skin and brain tissue (n = 594, AUC = 0.83–0.96) and thus represents a biomarker for biological age. Using the Uppsala Longitudinal Study of Adult Men birth-cohort (n = 108) we demonstrate that the RNA classifier is insensitive to confounding lifestyle biomarkers, while greater gene score at age 70 years is independently associated with better renal function at age 82 years and longevity. The gene score is ‘up-regulated’ in healthy human hippocampus with age, and when applied to blood RNA profiles from two large independent age-matched dementia case–control data sets (n = 717) the healthy controls have significantly greater gene scores than those with cognitive impairment. Alone, or when combined with our previously described prototype Alzheimer disease (AD) RNA ‘disease signature’, the healthy ageing RNA classifier is diagnostic for AD.

**Conclusions:**

We identify a novel and statistically robust multi-tissue RNA signature of human healthy ageing that can act as a diagnostic of future health, using only a peripheral blood sample. This RNA signature has great potential to assist research aimed at finding treatments for and/or management of AD and other ageing-related conditions.

**Electronic supplementary material:**

The online version of this article (doi:10.1186/s13059-015-0750-x) contains supplementary material, which is available to authorized users.

## Background

It is anticipated that novel genomic diagnostics that predict future health risks will help guide targeted preventative measures and enable the evaluation of individualized treatment strategies for many prevalent diseases of older age. So far, use of individual molecular biomarkers in healthy populations has offered modest performance [1, 2] compared with traditional, more integrated disease markers (e.g., blood pressure) or chronological age [3]. For example, in people with cardiovascular disease, circulating cystatin C concentration, a parameter that estimates renal function, was related to 10-year mortality but was insufficient to predict cardiovascular deaths in *healthy* older subjects [4]. Global RNA [5–9] and DNA methylation profiling [10–12] have been recently utilized to study the biology of chronological age. These existing signatures will incorporate influences of age-related disease and drug treatment. For example, Hannum et al. and Horvath et al. built distinct multi-tissue linear models, fitting age-related changes in DNA methylation with chronological age [13, 14]. These models have a statistical association with long-term health in the elderly [15] but the associations are not substantive enough to make it a practical diagnostic. In fact, as there are no molecular diagnostics of ‘healthy’ ageing status in humans, we hypothesized that a molecular profile may be useful at distinguishing people at risk for a variety of age-related diseases.

The shift in population demographics in the coming decades will mean that more than 1.2 billion people will be aged 65 years or older worldwide [16]. Approximately 7 % of this population will have dementia, with at least 60 % of these having Alzheimer’s disease (AD). AD is the single largest healthcare cost [17] and there are currently no drug treatments that halt or cure it [18]. Consensus is that only the earliest possible intervention is likely to significantly impact on AD and thus we need to identify those at greatest risk. The available validated diagnostics for AD are neither scalable for mass population screening nor sufficiently cost-effective to be practical [19]. For example, brain imaging can provide clear evidence of neurodegeneration but is restricted to specialist centers [20] and an imaging-based public health screening program would not be affordable [19, 21]. There is a pressing need to stratify the older healthy population, using simple and cost-effective methods, to, for example, identify those appropriate to enrich clinical trials of novel AD treatments. Prototype blood diagnostics can be 75–85 % accurate at distinguishing AD patients from controls; however, these have not been validated using independently processed samples or have failed to replicate in independent studies [20]. For example, blood-based protein signatures can diagnose mild cognitive impairment (MCI) and/or AD from controls in single studies [22–26], yet a common set of proteins has not been found across multiple studies. Further, the candidate AD marker proteins included cytokines and other markers of metabolic or cardiovascular disease [27] and thus these will not be *clinically* specific for AD when applied to older populations [28].

The expression of RNA is under genetic [29, 30], epigenetic [13] and environmental control [31, 32] and so the abundance of individual RNA molecules in blood cells reflects the integration of a variety of influences, whether or not blood directly interacts with a diseased organ. Thus, blood RNA [33–41] has been used to distinguish controls from MCI and/or AD, where variations in blood RNA expression should reflect the shared genetic, epigenetic [13] and environmental influences with the brain. For some prototype RNA diagnostics, the performances reported have been remarkably high (~95 %), but the same samples have been used during model building and validation [37, 38] and thus these represent examples of extreme over-fitting. In general there is always a danger that a classification model, when built using a specific set of cases and control samples from a single study, reflects unknown specific features of that particular cohort and thus is not generalizable.

In the present study we developed a RNA classifier of ‘healthy’ ageing starting with human muscle, with the hypothesis that this gene expression pattern may provide reliable genomic predictors for risk of age-related disease. We built the RNA classifier using human *muscle* global gene expression profiles because it has proven a useful tissue for predicting systemic physiological traits in humans [42] and because we can define healthy physiological status with ease [31]. When the RNA classifier was related to cognitive health, this ‘healthy ageing gene score’ had the advantage of being hypothesis driven, and built using a paradigm and samples entirely distinct from clinical case–control samples. When applied to blood RNA, we established good validation for AD diagnosis and selectivity over common age-related pathologies. The results of the present study further support the idea that analysis of peripheral blood RNA would be a fruitful strategy for developing biomarkers of cognitive health and prove that a common healthy ageing gene-expression program is detectable across multiple tissues.

## Results and discussion

### Identification of a reproducible RNA signature for age of human muscle, brain and skin

**Fig. 1:**
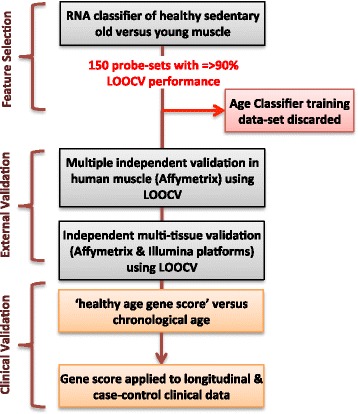
Development, validation and clinical application of ageing diagnostic. Overview of the selection process and use of RNA probe-sets for the development and validation of the healthy physiological age classifier. We identified useful probe-sets from a possible starting number of ~54,000 during step one [e.g. probe-sets with leave-one-out cross-validation (LOOCV) performance ≥ 90 %]. We then evaluated the performance of the top-ranked 150 probe-sets in a number of independent muscle, brain, and skin samples, demonstrating that the signature was diagnostic for age. We then applied the 150-probe-set healthy ageing signature to several clinical studies, as illustrated at the end of the workflow. Key features included discarding the training data set immediately after selecting the 150 probe-sets and relying on LOOCV and full external validation processes

Our objective was to discover a *pattern* of RNA expression that could be reliably used as a biomarker for ‘health status’ in older subjects — one that differed substantially in terms of ability to stratify health, and one that was more informative than chronological age. We applied machine-learning methods to RNA expression data to distinguish between healthy 25-year-old and healthy 65-year-old individuals. We took a simple classifier approach [43] without ad hoc *a priori* filtering to identify a consistent set of RNA markers of ageing across tissue types because standard differential expression is unable to provide a common multi-tissue set of discriminatory RNA molecules [9]. We selected muscle tissue gene-chip profiles from 15 sedentary young and 15 sedentary older subjects with good aerobic fitness (Gene Expression Omnibus (GEO) accession [GSE59880]) [31, 44] and who were free of diabetes [42, 44]. Specifically, we utilized a *k*-nearest neighbor (kNN) classification approach because this captures data features that share non-linear interactions with robust performance [45] and is a method consistent with strategies recommended by the Microarray Quality Control consortium [43]. This first data set — called the ‘training data-set’ — was used *only* once to select genes (Affymetrix probe-sets) and direction of gene expression change, and was then discarded from the project (Fig. 1). Expression differences of ~54,000 probe-sets were ranked using an empirical Bayesian statistic and a leave-one-out cross-validation (LOOCV) process (see “Materials and methods”). Probe-sets that targeted multiple genomic loci were removed and a 150 probe-set list, each gene having a nominal performance of 90 % or better, was selected for further study (Additional file 1). The extended list of probe-sets with a 70 % or better performance is also included in Additional file 1.

**Fig. 2:**
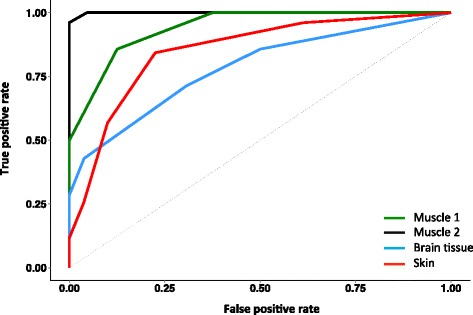
ROC curves showing predictive performance of the healthy ageing classifier based on LOOCV (kNN = 5) for muscle, brain, and skin. Using only the 150 probe-sets identified in the first stage of the project, this ‘healthy ageing classifier’ was able to correctly classify young and old samples across independent data sets with an accuracy of ~96 %, 91 %, 85 %, and 78 %. We present two examples of independent muscle data [48, 50] and one example each for human brain [50] and skin data [11] with areas under the curve of 0.99, 0.94, 0.78, and 0.85, respectively, reflecting excellent separation of the age groups and hence accurate multi-tissue performance

We checked that the 150 RNAs were not differentially expressed to any measurable extent in human muscle by exercise or a number of other common diseases that impact on skeletal muscle, using our previously published gene-chip data [8, 31, 44, 46]. We later confirmed this lack of association with lifestyle disease using a sensitive gene-set approach. Use of fully independent training and validation data sets allows for genuine external validation to be demonstrated (see “Materials and methods”). Using the ‘Campbell’ muscle data set [GEO:GSE9419] [47] as the samples of known identity, we demonstrated that additional young and old muscle samples selected from four additional muscle data sets (‘Trappe’ [GEO:GSE28422] [48], ‘Hoffman’ [GEO:GSE38718] [49], and ‘Kraus’ [GEO:GSE47969] and ‘Derby’ [GEO:GSE47881] [8]) could be classified with an average ~93 % accuracy (70–100 %) using only the 150 probe-sets selected at the start of the project. Substitution of the Campbell data set with the other muscle data sets worked equally as well. These data shared a common microarray platform (Affymetrix HGU133plus2) but, as we demonstrate below, the classifier remains robust in the face of alternative platforms. Receiver operating characteristic (ROC) curves for kNN = 5 demonstrating classifier performance for a number of tissue types are presented in Fig. 2.

Remarkably, the muscle-derived 150 RNA profile performed very well in classifying brain tissue by age. Using data from the HGU133Plus2 microarray platform for old and young samples of ectodermal origin (I, e, brain, n = 120) [50] we confirmed that the 150 RNA ‘healthy ageing’ genes selected in muscle could also distinguish the age of human brain one sample at a time, with a classification success rate up to 91 % (Fig. 2). Four brain regions were evaluated (postcentral gyrus, entorhinal cortex, hippocampus and superior frontal gyrus; [GEO:GSE11882]) and while they were confirmed disease-free by histopathology in the original study [50], unlike our muscle cohorts, their true functional status remains unknown. The postcentral gyrus samples were classified with 86 % sensitivity and 89 % specificity. In this cohort, older hippocampal regions were often misclassified using the 150 genes (33 % sensitivity) as ‘young’. This higher misclassification rate may relate to the substantial neurogenesis known to take place in the adult hippocampus or delays in tissue processing. We evaluated whether the 150 genes could accurately classify the age of tissue of mesodermal origin (skin) using gene expression data in a total of 279 human skin samples, of which there were up to three technical replicates per clinical sample [9]. Notably, these data originated from a different technology platform (Illumina Human HT-12 V3, Array-express: E-TABM-1140), adding variability above that derived from a distinct tissue and potentially limiting the classification process. The two gene-chip technologies had 129 genes in common, and we observed excellent classification of human skin age [n = 131, area under the curve (AUC) = 0.85; Fig. 2]. The classification success was similar for all three replicates (71–78 raw classification success). Thus, the technical performance of the 150-gene healthy ageing classifier was excellent, providing accurate tissue classification despite inter-laboratory technical variation, different gene-chip platforms and antemortem issues. We were able, therefore, to conclude that we have identified a reliable multi-tissue RNA signature of healthy tissue ageing in humans, something that has not been previously demonstrated [8, 9].

### A healthy ageing gene score that is distinct from chronological age and unrelated to lifestyle regulated phenotypes in the ULSAM study

**Fig. 3:**
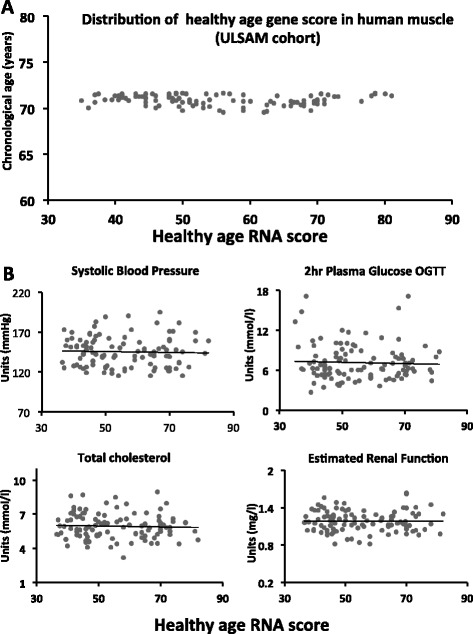
Distribution of healthy ageing gene score in ULSAM samples and its relation with clinical parameters. At the date of assessment (1992), when the muscle biopsy was taken for subsequent gene-chip profiling, all subjects were considered in reasonable health for their age and remained physically active. **a** Distribution of gene score based on the median rank for each of the 150 genes (see “Materials and methods”). **b** Clinical variables were determined as previously reported for ULSAM samples (chronological age = 69–70 years) [71, 101]. Linear regression was used to examine the relationship between the healthy ageing gene score at ~70 years and a variety of clinical parameters at age ~70 years. No relationship between baseline gene score and renal function (estimated from cystatin C, r2 < 0.001), systolic blood pressure (mmHg, r2 = 0.0013), 2 h glucose concentration following a standard oral glucose tolerance test (*OGTT*; mmol, r2 = 0.015) or total cholesterol (mmol, r2 = 0.002) was observed. Gene score was also unrelated to resting heart rate or physical activity questionnaire, and thus habitual exercise status. In fact the healthy ageing gene score was not correlated with any conventional risk factors (as listed in Additional file 2)

In order to examine specificity for ‘healthy ageing’, we examined the relationship between the classifier genes, chronological age and markers of lifestyle-associated genes. We collapsed the expression pattern of all genes into a single score for each sample (see “Materials and methods”). The distribution of scores was examined for ~70-year-old males (subjects born in Uppsala within a 1-year period) and the gene ranking score was also correlated with markers of lifestyle-associated disease (Fig. 3). The gene expression profiles from 108 muscle samples from ~70-year-old male subjects from the Uppsala Longitudinal Study of Adult Men (ULSAM) cohort [51] were produced using Affymetrix arrays (Human Exon 1.0 ST Array). We ranked each subject for each of the 150 genes, taking the direction of gene expression change from the original classifier model into account (85 % down-regulated; see “Materials and methods”). We then converted the individual gene rankings into a summed median gene score for each subject. We demonstrated that despite all subjects being ~70 years of age at the time of the RNA sample, there was a very wide distribution in gene score (Fig. 3a). Thus, the healthy ageing gene score in muscle was very distinct from chronological age. The healthy ageing gene score was regressed against a variety of continuous clinical variables (variables listed in Additional file 2). The gene score at chronological age ~70 years was unrelated to conventional lifestyle regulated biomarkers (e.g., blood pressure, glucose, cholesterol, or renal function; Fig. 3b). This confirmed that the 150 gene expression markers were not reflecting a variety of lifestyle regulated biomarkers and diseases (e.g., exercise, diabetes) and tissue ‘healthy ageing status’ could not be derived from a simpler clinical biomarker.

Despite the limited sample size of the ULSAM cohort (n = 108), we were also able to demonstrate that subjects with the highest muscle healthy ageing gene score at age 70 years had significantly better renal function 12 years later (at age 82 years, *p* = 0.009). Remarkably, the healthy ageing gene score in muscle at ~70 years was also independently related to 20-year survival (*p* = 0.0295; Fig. S1a in Additional file 3) in a logistic regression model that included factors listed in Additional file 2). While this observation should be interpreted cautiously, to illustrate the temporal relationship between the healthy ageing gene score and death, we divided the gene score into quartiles and applied a Cox-regression model (Fig. S1b in Additional file 3) and found a significant difference between the first versus the fourth quartile (*p* = 0.04). In contrast to the healthy ageing gene score, a median gene rank score based on inflammatory gene (GO:0006954) or mitochondrial gene (GO:0005739) expression in muscle demonstrated no relationship with health or mortality (data not shown). The significant relationship between the healthy ageing gene score and organ function demonstrates that the gene expression pattern most similar to the healthy 65-year profile in the classifier model (i.e., the largest gene score in the ranking system) was associated with better health in the ULSAM cohort.

### A greater healthy ageing gene expression score is associated with better cognitive health

**Fig. 4:**
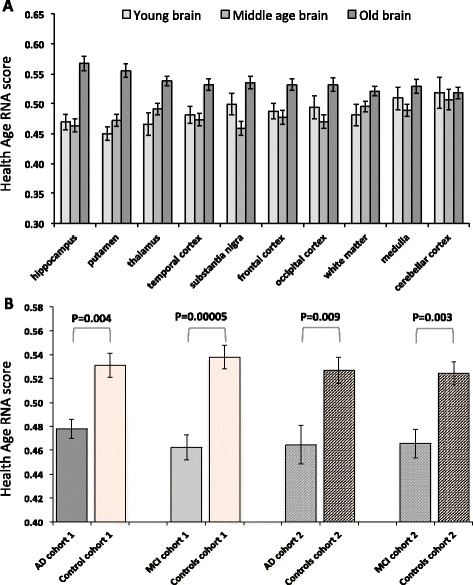
The healthy ageing RNA signature in healthy human brain tissue and blood of AD patients and controls. There was robust regulation of the healthy ageing RNA signature in human brain with healthy ageing and between control subjects and subjects with AD or MCI. **a** The healthy ageing RNA signature was studied across brain regions in healthy individuals using BrainEac.org gene-chip resource [GEO:GSE60862]. Ten brain regions from 134 subjects representing 1231 samples were individually ranked (see “Materials and methods”) and the median sum of the ranked scores calculated. Regulation of the healthy ageing genes differed across brain regions with age, as determined by a Kruskal Wallis Test (hippocampus *p* = 0.00000002, putamen *p* = 0.00000004, thalamus *p* = 0.00004, temporal cortex *p* = 0.0001, substantia nigra *p* = 0.0002, frontal cortex *p* = 0.001, occipital cortex *p* = 0.001, white matter *p* = 0.01, medulla *p* = 0.06 and cerebellar cortex *p* = 0.51). Post hoc Mann–Whitney test, with correction for multiple comparisons (Holm), confirmed a striking ‘increase’ of the healthy ageing score in the healthy older samples (hippocampus, putamen, thalamus, substantia nigra, and the occipital, frontal, and temporal cortex regions; at least *p* < 0.002). **b** The healthy ageing RNA signature was studied in blood samples from two independently processed case–control studies of AD. In cohort 1 the control median gene score was greater (*p* = 0.004) than AD samples and greater (*p* = 0.00005) than that of the MCI samples (Wilcoxon rank sum test). In cohort 2 the median gene score of control samples was greater than that of AD samples (*p* = 0.009) and that of MCI samples (*p* = 0.003). Data are median gene score and standard error

Neurocognitive pathology (e.g., AD) becomes more pronounced with age and is often apparent in individuals who are otherwise healthy. Our analysis of the relationship between lifestyle factors and the healthy ageing gene score in the ULSAM cohort suggested that the gene score was robust to confounding effects of lifestyle disease. We next examined whether the healthy ageing gene score (median rank sum of the 150 RNA markers) was *selectively* useful in relation to identifying neurocognitive disease over lifestyle disease. To support this analysis, we utilized a large publically available gene-chip data set derived from healthy human brain samples of various ages [52]. The BrainEac.org gene-chip resource [52] [GEO:GSE60862] comprises ten post-mortem brain regions from 134 subjects representing 1231 samples (Additional file 1). Using the same ranking approach as applied to the ULSAM cohort, the median sum of the rank score was calculated for each anatomical brain region (Fig. 4a). As before, in healthy older individuals the ‘age’ signature was ‘switched on’ (yielding a greater ranking score) compared with younger subjects. Regulation of the healthy ageing gene score increased in a distinct manner across individual healthy brain regions with chronological age, especially in the hippocampus (*p* = 0.00000002), as well as other regions (putamen, thalamus, substantia nigra, and the occipital, frontal, and temporal cortex regions (all at least *p* < 0.002 by Holm adjusted Mann–Whitney test).

Our primary hypothesis was that, compared with control subjects of similar chronological age and gender, patients with AD would have a lower median healthy ageing gene score, but the score would not distinguish diabetes or vascular (i.e. lifestyle influenced) disease patients from matched controls. We used two independent case–control studies of AD and two case–control studies of lifestyle disease with RNA profiles derived from blood. The first AD cohort has been previously used to study disease pathway changes in blood [41, 53] and we have deposited this data set (cohort 1 [GEO:GSE63060]) and a second analysis (cohort 2 [GEO:GSE63061]) at the GEO. We first used a maximum possible subset of subjects from each entire cohort, so that gender and chronological age could be precisely balanced (~70 years) remove these as potentially confounding factors. From cohort 1, 113 subjects were ranked for gene score, while 111 subjects were ranked in cohort 2 (Table 1). We checked for overlap between the 150 healthy ageing gene markers and previous genomic and genetic disease markers of AD (Additional file 1). Only three genes were in common and none were from previously validated AD diagnostics. Their inclusion or exclusion did not impact our analyses.

**Table 1:** Clinical characteristics of batch 1 and batch 2 of case–control subjects that contributed to the blood gene-chip profiles analyzed and presented in Figs. 4 and 5

Gender and age-matched cohorts	Age	Gender (F/M)	MMSE	CDR-SOB
Batch 1				
Control_MCI_ (n = 67)	69.6 (±4.2)	41/26 (61 % F)	29.1 (±1.2)	0.07 (±0.18)
MCI (n = 39)	70.0 (±3.3)	24/15 (62 % F)	27.5 (±1.6)	1.24 (±1.60)
Control_AD_ (n = 64)	70.2 (±3.7)	41/23 (64 % F)	29.1 (±1.2)	0.08 (±0.18)
AD (n = 49)	69.8 (±4.4)	34/15 (69 % F)	21.8 (±4.5)	5.44 (±2.95)
Batch 2				
Control_MCI_ (n = 71)	70.8 (±2.9)	44/27 (62 % F)	28.9 (±1.9)	0.15 (±0.57)
MCI (n = 31)	69.5 (±4.5)	23/8 (74 % F)	27.6 (±1.9)	1.34 (±1.86)
Control_AD_ (n = 71)	70.8 (±2.9)	44/27 (62 % F)	28.9 (±1.9)	0.15 (±0.57)
AD (n = 40)	69.9 (±4.3)	23/17 (58 % F)	21.0 (±5.6)	5.80 (±2.75)

The subjects are an age- and gender-balanced subset of the entire clinical cohort. *MCI* mild cognitive impairment, *AD* Alzheimer’s disease. Age is in years (±standard deviation). Gender is ratio of females (*F*) to males (*M*). *MMSE* mini-mental state examination involving a 30-point questionnaire. *CDR-SOB* the Washington University Clinical Dementia Rating Scale (CDR) global and Sum of Boxes (SOB) score. Application of the healthy gene ranking score provided, post hoc, similar separation of the groups with similarly robust statistical significance

Blood RNA from AD case–control cohort 1 was profiled on Illumina HT-12 V3 bead-chips. We first mapped the appropriate probes from Affymetrix to Illumina, yielding 128 genes from the original 150-gene list. The relative median rank score for AD patients was significantly lower than for the age- and gender-matched controls (*p* = 0.004; Fig. 4b) based on Wilcoxon rank sum test. Blood RNA from the second AD case–control cohort was profiled on the Illumina HT-12 V4 platform and in this case 122 genes were in common with the 150-gene healthy ageing gene signature. As before, the median rank healthy ageing gene score for AD patients in cohort 2 was significantly lower than in the control group (*p* = 0.009; Fig. 4b). Furthermore, for both cohort 1 and cohort 2, the age-matched controls had a higher median gene score than subjects diagnosed with MCI (Fig. 4b; *p* = 0.00005 and *p* = 0.003 for cohorts 1 and 2, respectively). It is important to note that the control samples used for comparison with MCI overlapped with those used for comparison with AD and that the MCI analysis cannot, therefore, be considered a fully independent observation. As expected from the ULSAM analysis, the healthy ageing gene score was not related to diabetes or vascular disease status using blood profiles from 366 individuals (Additional file 4).

**Fig. 5:**
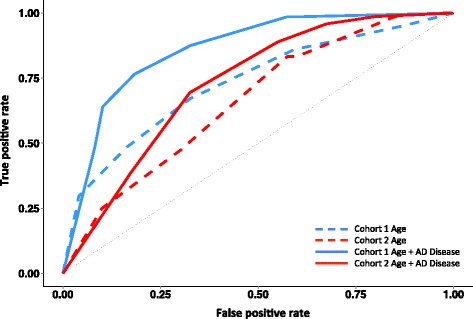
Validation of novel blood RNA classifiers as a diagnostic for Alzheimer’s disease. We used the independent batch 2 AD data set (see “Materials and methods”) to test the predictive performance of our healthy ageing classifier and our previously published AD prototype diagnostic. The performance of each was evaluated using ROC curves. The healthy ageing gene classifier generated independent AUCs of 0.73 and 0.66 for AD in cohorts 1 and 2, respectively. For the combined ‘healthy ageing’ plus ‘AD disease’ RNA classifier (150 + 48 probe-sets) we obtained AUCs of 0.86 and 0.73 for AD without *any* attempt at optimization. The AD disease RNA classifier probe-sets were selected using cohort 1

We formally evaluated whether the healthy ageing signature could act as a diagnostic for AD using ROC analysis and found that it had robust independent performance (AUC = 0.66–0.73; Fig. 5). We have previously published a whole blood RNA-based prototype AD diagnostic [41] consisting of 48 genes identified using machine learning methods applied to cohort 1 samples. We demonstrated that this prototype ‘RNA disease signature’ was independently validated in cohort 2 using LOOCV. Further, when we combined the two independently produced and validated gene expression classifiers we yielded an improved AD diagnostic (AUC = 0.73–0.86; Fig. 5) that matches best in class [54] for blood-based AD diagnostics validated using independent data, while our RNA-based analysis uses a technology platform more suited to reproducible high-throughput diagnostics.

#### Biological features of the healthy ageing diagnostic

**Fig. 6:**
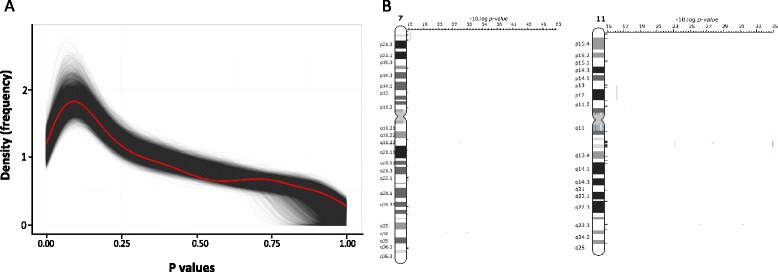
Gene ontology profile and chromosomal positional enrichment analysis. Pathway analysis and GO analysis indicate that the 150 healthy ageing genes are not related to a few specific biological processes but rather originate from across many biological processes. **a** Density curves of raw *p* values for each of the 10,000 hypergeometric tests using randomly sampled probe-sets from the U133+2 gene-chip (n = 150 each time; *black*) and the density curve of the raw *p* values from a hypergeometric test using the 150 healthy ageing gene classifier probe-sets (*red*). A similar result was obtained when the top 670 genes were utilized as the input and compared with randomly generated gene sets of 670 genes. **b** Positional gene enrichment analysis for the top 670 genes from the prototype classifier (670 probes from which the top 150 probes, with performance >90 %, were selected) found over-representation at 7q22, 11q13 and 11q23. Results were consistent using positional gene enrichment analysis and the ToppGene algorithm; both identified 3, 12 and 3 genes at each loci, respectively, with *p* < 0.001 or less. Those for 11q13 and 11q23 in particular were most significant, and contained genetic variants that influence the age of onset of various cancers

We were interested in whether the healthy ageing diagnostic identified any particular biological processes that might be open to therapeutic targeting. The 150-gene list (Additional file 1) was evaluated using both Ingenuity pathway analysis and R-based gene ontology (GO) analysis. Ingenuity analysis (where a total of 127 genes were annotated in the database) revealed a few marginal functional associations (e.g., nervous system development genes) but these did not remain significant following Benjamini and Hochberg correction. The top ranked database network (genes with published interactions) was defined as ‘cell death and survival’ and contained 31 molecules. In Fig. 6a the density curves of *p* values for each one of 10,000 hypergeometric tests using a randomly sampled gene set (n = 150 in size) are plotted (black), along with the density curve of the *p* values from the healthy ageing 150-gene set (red). The profile of ontological enrichment in the healthy ageing diagnostic was not different from a random sample of 150 genes from the gene-chip, of which more than 99 % of the 54,000 probe-sets had no ability to discriminate tissue age in our training model. Manual searching of PubMed and the Online Mendelian Inheritance in Man (OMIM) database yielded some plausible connections with age-related and disease processes (Additional file 1) but such analysis is subjective and it cannot be concluded that these biological functions appear in the ‘healthy ageing’ diagnostic more than by simple proportionality. We did note that the 150 genes included some previously identified ‘ageing’ genes, for example, *LMNA* (linked with Hutchinson-Gilford progeria syndrome), Unc-13 homolog (*UNC13C*; linked with beta-amyloid biology), as well as *COL1A1* (thought to change in skin ageing).

We also examined whether the 150 age-related genes were over represented at genomic loci using positional enrichment analysis [55] but found no significant associations. Using the top 670 genes from the first stage of the project (>70 % success in the training model) there were a number of significant findings (Additional file 1) with three genes originating from the top 150. In this analysis, 11q made a significantly greater contribution (adjusted *p* value = 0.005–0.007) to the enlarged prototype classifier than would be expected by chance (Fig. 6b), and there were a total of 15 genes from the 11q13 (*ALDH3B1*, *CAPN1*, *CDC42EP2*, *CORO1B*, *LTBP3*, *NRXN2*, *PPP1R14B*, *RCE1*, *RCOR2*, *SART1*, *SYT12*, and *ZDHHC24*; *p* = 0.0005) and 11q23 (*FXYD2*, *SCN2B*, and *TMPRSS13*; *p* = 0.0009) over-represented genomic locations. Interestingly, 11q23 is the location for age-related genetic interactions, namely the apolipoprotein A family [56, 57] as well as a region containing genetic association single nucleotide polymorphisms (SNPs) which modify the age of onset of colorectal cancer [58, 59]. Furthermore, 11q13 harbors SNPs associated with age of onset of renal cell carcinoma and prostate cancer and modulating age-related disease emergence by 5 years [60–62]. While the lack of an apparent specific biological dialogue may be considered disappointing, the extensive independent clinical results strongly support that the novel 150-gene healthy ageing signature is an important marker of healthy ageing in humans. Therefore, regulation of this gene expression program may in time reveal itself to be an important mechanism for maintaining human health and thereby a new opportunity for target development.

The molecular mechanisms that define healthy ageing remain elusive in both human and animal models [63]. Many of the molecular mechanisms which extend the lifespan of laboratory animals also appear to extend health-span or disease-free ageing in these models [64]. However, it remains unclear whether any of these mechanisms are central to human ageing [8, 9, 65] or define healthy ageing in humans. Our approach was novel because we first sought to define a set of genes associated with healthy ageing in ‘normal’ 65-year-old subjects rather than gene expression associated with disease or extreme longevity. This is an important distinction — ageing is thought to be a continuous physiological process that could be expected to have a gene expression signature distinct from lifestyle related (e.g., type II diabetes) or mutation driven (e.g., cancer) pathologies, thus explaining its independent prognostic rather than specific diagnostic capacity. It is however of potential importance that regulation of the healthy ageing signature in human brain is most evident in those regions associated with neurodegeneration. In contrast, it is thought that the cerebellar cortex is not subject to substantial age-related anatomical changes [66] and this was consistent with our new model of human healthy ageing (Fig. 4a).

The 65-year-old subjects used to build the RNA model were in good health despite leading a normal sedentary lifestyle. Rather than using individual differential expression values to define discriminatory genes, we selected a group of genes that would act together to make a ‘majority vote’. Indeed, we were able to demonstrate that the 150 healthy ageing genes are consistently modulated in several tissue types, but to very differing degrees in people of the same chronological age (e.g., Fig. 3a). Thus, the healthy ageing gene score fulfilled the first main criteria for being a novel diagnostic of healthy (or biological) ageing. Including the ULSAM analysis (males only), we have demonstrated in three independent clinical cohorts that greater healthy ageing gene score associates with better health in men and women, suggesting that promotion of this gene expression profile may be beneficial and could reflect an adaptive compensatory response. The present RNA diagnostic could be used to facilitate the evaluation of anti-ageing-related treatments in middle-aged humans, screen for long-term safety during drug development, or augment clinical decision-making that currently inputs chronological age rather than ‘biological’ age into treatment algorithms. Future efforts should focus on discovering strategies to modulate the healthy ageing gene signature to establish if it is causally determining health or just acting as a robust biomarker of a more complex set of molecular interactions.

### The multi-tissue healthy ageing gene score is predictive of health in older subjects

Exceptional longevity is driven by a measurable genetic contribution [67, 68], while being active and healthy at age 65 years is a more common occurrence, likely to reflect complex molecular factors [64, 69], and is less obviously linked to only variations in DNA sequence. We profiled RNA from healthy members of the ULSAM cohort at age 70 years and analyzed follow-up data over two decades. In 1992, these 70-year-old Swedish men had normal levels of physical activity ‘for their age’ and most demonstrated longevity to 90 years, which is not exceptional in the Swedish population [70]. The healthy ageing score demonstrated a *four-fold* range (Fig. 3a) while chronological age varied by no more than one year across the group. A greater gene score was associated with better cognitive function, and better renal function across a 12 year span and both cognitive decline and renal function are important determinants of all-cause mortality [71, 72]. A concurrent reduction in cognitive and renal function is clinically observed, suggesting both are subject to a general age-related decline in organ function [73]. It is perceived that lifestyle-regulated diseases, such as type II diabetes, causally increase AD [74]. This relationship did not appear causal when the rates of emergence of diabetes (or not) and number of emerging cases of AD were compared in the Framingham or Baltimore Age studies [75, 76]. This suggests that risk of developing type II diabetes and AD may share some concurrent risk factors, e.g., aerobic capacity [77]; a physiological capacity defined by a large genetic and gene–environmental interaction [78]. Additionally, type II diabetes and AD may share epigenetic or genetic risk factors. Interpretation of such associations is further complicated by the interaction between type II diabetes, vascular disease, and other types of dementia (which complicate the diagnosis of AD).

Neurological decline is predicted to contribute substantially to the economic burden of healthcare in the coming decades. AD is a multi-factorial disease [79] with around 22 genetic loci *potentially* associated with disease risk or progression of symptoms. The strongest and most reproducible genomic association, APOE-ε4, is a modifier of risk, contributing to the variance in age of onset of the disease by 3.7 % [80]. The remaining approximately nine reproducible risk loci for late-onset AD (the most common form) contribute a further 2.2 % of the variance in age of onset [80]. In short, these DNA sequence variants will not be clinically useful for diagnosing or managing AD or even assessing risk in the majority of people. Differential gene expression analysis and molecular classification have found disease-related RNA markers of AD, using patient materials to build the model [35]. However, such diagnostics can be biased by unknown features of the training data (the data used to select the RNA markers). In contrast our healthy ageing genes were selected via a hypothesis-driven strategy that then relied on a validation process that included seven independent tissue cohorts including multiple RNA detection technologies (so ruling out some unknown technology platform bias). Thus, our healthy ageing gene expression signature has the key advantage of being a signature built using a paradigm and samples entirely distinct from AD case–control samples.

The healthy ageing gene score allowed us to demonstrate that patients diagnosed with AD have an altered healthy ageing RNA expression signature in blood that demonstrates significant association with disease. Furthermore, the muscle or blood gene score was unrelated to lifestyle diseases such as type II diabetes and thus may be more clinically specific than earlier AD biomarkers [20, 22–26, 41, 81], most of which did not replicate in independent clinical studies. We were able to provide independent validation for our earlier AD-related ‘disease’ diagnostic [41]; like many AD disease biomarkers [35], however, it includes pro-inflammatory markers and oxidative stress, features that can be common to several diseases and thus it may not be specific in clinical practice. Nevertheless, when we combined the Lunnon et al. [41] AD biomarker (even after removing the eight genes we found to be regulated in blood by diabetes or vascular disease) with the healthy ageing genes we yielded an improved diagnostic for AD over and above either diagnostic alone (Fig. 5). Ultimately, formal diagnosis of AD will continue to rely on a combination of diagnostics, including invasive cerebrospinal fluid sampling, positron emission tomography (PET) imaging and magnetic resonance imaging (MRI). However, given the scale of screening required (e.g., more than 1 million people in 2015/2016 to deliver sufficient numbers of at-risk subjects for AD clinical trials [82]) a blood-based diagnostic will be extremely useful for pre-screening ahead of invasive and costly follow-up analysis. Enrichment of prevention trials with asymptomatic people most at risk for AD is required to ensure that event rates are sufficiently high to evaluate the multitude of drug trials being considered for AD [20].

Like many genomic diagnostics, the full clinical utility of ours will only emerge when combined with additional data and clinical insight. While we could also demonstrate that patients with MCI had a significantly lower healthy ageing gene score it remains to be shown that this can be converted into a diagnostic for future cognitive health (i.e., a blood sample from older healthy subjects or those with recently diagnosed MCI combined with 5–10 years of follow-up data to prove they did or did not develop AD). Epidemiological efforts to build long-range (~36 year) forecasting of dementia risk (AD or vascular) using clinical demographics [83] (CAIDE score) provide assessment of risk at middle-age (~45 years and over) and can assign patients into a low (9 %) or high (29 %) risk with *un-validated* ROC AUC = 0.74. In diabetes patients, age is by far the more powerful predictor of future dementia rather than severity of the diabetes measured using glycosylated hemoglobin A1 (HbA_1_) [84] and it will be informative to replace age with our healthy ageing gene diagnostic for many conditions. These examples highlight that, clinically, various decision trees exist and our healthy ageing score could be integrated to help decide which middle-aged subjects could be offered entry into a preventative clinical trial many years before the clinical expression of AD.

As can be observed in Fig. 5, we obtained an independently validated ROC AUC of 0.73 using default classification settings. This is not an optimized or over-fitted model, in that it is most likely possible to tune ROC parameters to yield an improved performance using the same list of genes or a subset thereof. Some previous authors have reported ROC AUC scores of >0.8, but as mentioned these do not represent valid scores, being derived from data over-fitted to a single data set [33–40]. High ‘scores’ encompass shared technical variance in the ‘test’ and ‘validation’ data. For example, a microRNA dementia diagnostic (relying on 2–17 microRNA real-time PCR assays) was validated in samples used to build the initial model, yielding inflated specificity and sensitivity values [40]. Other practical factors must be considered, such as the complexity of the laboratory test and costs. Cheng et al. [39] used a complex process to isolate serum exosome microRNAs and a split-cohort partial validation approach. Their best AD model (16 microRNAs) was 87 % sensitive and 77 % specific but could not diagnose MCI. DiaMir Inc. claimed 95 % specificity and sensitivity for MCI (their model did not work in AD) but this failed to replicate in a second study [40].

During the past 5 years several projects have worked with whole-blood mRNA and produced 20–225-gene assays to classify samples during the training phase, comparing controls with patients (MCI or AD). The RNA classifier from DiaGenic A/S used TaqMan assays and this remains the only replicated blood-based AD diagnostic performing to a similar level as the present study [33], while there were no genes in common with our own ‘AD disease’ or ‘healthy ageing’ RNA signatures. To date, few blood-based protein and metabolite diagnostics have been replicated using a fully independent process. Doecke et al. [27] combined a panel of eight protein markers with age, gender, and APOE genotype in the Australian Imaging, Biomarkers and Lifestyle (AIBL) cohort and found an AUC of 0.84 using a subset of the Alzheimer's Disease Neuroimaging Initiative (ADNI) cohort. However, their protein markers were *a priori* known to be regulated in the ADNI cohort, and it remains unclear how many of the eight protein markers contribute to the model, including age, gender, and genotype, in the validation process.

### Comparison with known markers of human ageing or longevity

Other approaches have been utilized in humans to understand the molecular determinants of human ageing, but not ‘healthy’ ageing. Genome-wide association analysis has shown 281 DNA variants linked with exceptional longevity, and collectively explaining 17 % variance in humans [68] with an AUC value of 0.6. This remains to be replicated and this list of genes did not overlap with our healthy ageing gene list. In addition, long-lived humans appear to have a similar genetic burden for common DNA disease variants, suggesting the human exceptional longevity model may not be reflective of the processes that determine average longevity [63]. There have been several linear molecular models of chronological age [5, 8, 11, 14] but the variance captured by these across chronological age is limited and the disease status of samples used to build or validate the model unclear; thus, it is uncertain if such models reflect ageing or age-related disease and drug treatment. There was no overlap between the genes in our healthy ageing RNA classifier and the quasi-linear methylation model derived by Horvath et al. [13].

## Conclusion

We found four genes in common between our healthy ageing RNA classifier and the two gene lists identified by Hannum et al. in separate DNA methylation models of ageing (n = 94 and n = 326): one gene from their primary model (*PKM2*) and three genes from their RNA methylation association analysis (*ANKRD13B*, *RUNX3*, and *TCF3*) [14]. Neither the Horvath nor the Hannum models generate sufficient distinction from chronological age to provide a useable ‘size effect’ when considering longevity [15]. Passtoors et al. [5] reported that a set of 21 RNA molecules ‘marked out’ familial longevity in blood RNA, but this was a weak correlation with no discriminatory capacity as a diagnostic, possibly because it reflects a mixture of ageing, disease, and drug therapy. Furthermore, none of those age-related blood RNA changes were consistently correlated with age in human brain or muscle [8, 85], indicating that these 21 RNAs do not represent universal markers of human ageing (they were also not part of our 150 healthy ageing gene list). We did not note any significant ontology pathway enrichment within our healthy ageing diagnostic gene lists (Fig. 6a). Thus, we cannot neatly place the genes that contribute to the healthy physiological age diagnostic into a convenient canonical signaling pathway.

## Materials and methods

Informed consent was obtained from all volunteers and ethical approval received from Institutional Research Ethics Committee as reported in primary clinical publications [8, 9, 31, 44, 46–50, 52] and all studies included in this work were conducted under the auspices of the declaration of Helsinki. For new gene-chip tissue profiles and hence new GEO deposits, the Institutional Research Ethics Committee approvals were as follows: ULSAM (Regional Ethical Review Boards Uppsala Ethical applications 09-154M/2010/400), STRRIDE (Duke Medical School IRB, Pro00012628) and AddNeuroMed/DCR (SLaM/IOP 30/07/2006/SLaM/IOP 30/04/2008).

A summary of the analysis strategy was as follows. The first aim was to generate a reliable RNA classifier of healthy older muscle tissue (healthy ageing gene score). We utilized k-nearest neighbor (*k*NN) classification methods because they capture data features that share non-linear interactions and have robust performance using methods consistent with the Microarray Quality Control Consortium [43]. The probe-set level intensities of each set of independent microarrays were normalized using the Robust Multi-array Analysis (RMA) method implemented within the R statistical software environment using the ‘affy’ package, and then scaled and centered (Bioconductor project [86, 87]). When Affymetrix gene-chips originated from independent laboratories, we used Frozen Robust Multi-array Analysis (fRMA) [88, 89]. Having identified a healthy ageing gene score comprising 150 RNA markers (probe-sets), we established that these 150 RNAs could reliably classify multiple *independent* sets of human muscle and brain tissue using external validation. External validation uses independent training and validation data sets. Finally, we examined if the healthy ageing gene score in blood was related to cognitive health, alone or in combination with our prototype blood marker of early AD. Figure 1 presents the project analysis scheme.

### Production and independent external validation of the healthy ageing gene score

We identified 150 RNA markers of muscle ageing using samples [31, 44] and gene-chip profiles [GEO:GSE59880] from 15 young (aged 19–28 years) and 15 older subjects (aged 59–77 years) free from metabolic and signs of cardiovascular disease and validated this observation in more than 500 independent samples. The older subjects were sedentary (did not do any regular sport/exercise) but nevertheless were free of diabetes and had good levels of aerobic capacity, a marker of general health into older age [90]. The RNA markers were selected using a nested-loop, holding out two arrays at any one time to estimate two parameters from the data. The first of these was the conventional classification result; i.e., was the ‘unknown’ sample correctly classified, yes or no? The second parameter was used to calculate the performance of the probe-sets contributing to the decision. We selected 200 probe-sets during each of the inner-most loops by ranking gene expression differences using an empirical Bayesian statistic (implemented as eBayes in the ‘limma’ package) [91]. Following iterative assessment of all probe-sets and all samples, involving ~180,000 permutations, a list of ~800 probe-sets was identified as having good performance (>70 % correct). We removed probe-sets that targeted multiple genomic loci and selected the top ranked 150 probe-sets (involved in >90 % correct decisions) for further study. Classifier performance was assessed using ROC analysis and the R package ROCR [92].

We implemented fully independent *external* validation of the 150-probe-set healthy ageing classifier, a process that requires both *independent* ‘known samples’ to define the expression space and *independent* test gene-chips [93]. When combined with LOOCV methods, this represents a gold standard approach to validation of a classification model. A new set of young and old muscle profiles (selected from the Campbell data set; n = 66 chips [47]; [GEO:GSE9419]) was used to represent the new expression space of known samples. We then carried out evaluation of sets of independent gene-chip profiles from young and old human muscle (all Affymetrix U133+2) normalized using fRMA. The various fully independent samples were obtained from GEO or produced from our own clinical samples [94]. For each dataset a subset of samples were selected to belong to either the young (~25 years) or older group (~65 years) from a larger collection of samples. The sets of young and older samples were selected from the Trappe [48] [GEO:GSE28422]; *n* = 48), Hoffman [49] [GEO:GSE38718]; *n* = 22), Derby [8] [GEO:GSE47881]; *n* = 26) and Kraus [GEO:GSE47969]; *n* = 33) data sets. For the Kraus data set total RNA was extracted from frozen muscle biopsy samples (*vastas lateralis*) using TRIzol reagent and *in vitro* transcription was performed using the Bioarray high yield RNA transcript labeling kit (P/N 900182, Affymetrix, Inc.) as previously described [95]. For all data sets, arrays were examined using hierarchical clustering and normalized unscaled standard error (NUSE). In cases where we identified a small number of gene-chips (two to three) that had evidence of technical defects, these were removed prior to any analysis.

To assess if human brain and skin also demonstrated the same 150 age-related gene expression signature as healthy older muscle, we used young and old samples from the brain-bank array source (n = 120; [GEO:GSE11882]) and the MuTHER cohort skin data set (n = 279, which includes a subset of three replicates, n = 131, n = 124, and n = 24). The skin data were produced using the Illumina Human HT-12 V3 Bead chip (Array-express: E-TABM-1140) and log-2 transformed signals were normalized using quantile normalization. The 150 Affymetrix probe-sets were mapped to the Illumina platform (giving 129 probes). Due to differences in gene-chip technology, a LOOCV approach was used to classify the age of each skin sample, using only the 150 probes selected at the start of the project. For skin, individuals aged 45 years or less were defined as young, and those aged 70 years or older as old to ensure balanced numbers of young and old samples existed to fairly assess the classifier performance. The three sets of technical replicates were analyzed separately and confirmed the intra-study technical reproducibility of the classifier using repeated RNA profiles of a single clinical sample (data not shown).

### The healthy muscle ageing gene score differs substantially from chronological age

We used a set of tissue samples from a birth cohort of men, such that the same chronological age (~70 years) could be contrasted with the variation in healthy ageing gene score. The ULSAM cohort comprises men born in 1920–1924 and living in Uppsala, Sweden and was used to compare a constant chronological age (and similar environment) with the healthy muscle age gene score across individuals [51]. Dual-energy X-ray absorptiometry (DXA) scan measurements were performed during the last decade of the study and muscle mass status varied between −15 % to +10 % between age 70 years and 88 years and was unrelated to physical activity scores (recorded at 82 years and 88 years of age, with 80 % being recorded as being moderately active). Renal function was estimated using cystatin C, which is a marker of glomerular filtration rate [4]. We had access to 129 skeletal muscle biopsies taken at age 70 years (in 1992) and we processed these in 2012 with the majority having excellent NUSE plot profiles. Total RNA was extracted from frozen muscle biopsy samples (vastas lateralis) using TRIzol reagent as previously described [95]. A total of 113 samples provided sufficient RNA and 50 ng total RNA was amplified using Ambion’s WT expression kit to produce cDNA. The cDNA was fragmented and labeled with GeneChip WT Terminal labeling kit (Affymetrix, Inc.). Unincorporated nucleotides from the *in vitro* transcription reaction were removed using an RNeasy column (QIAGEN Inc.). Hybridization, washing, staining, and scanning of the arrays were performed according to the manufacturer’s instructions (Affymetrix, Inc. Santa Clara, USA).

One hundred and eight samples passed gene-chip quality control procedures (see above). A cumulative gene ranking-based score was calculated using each of the 150 gene expression values for each of the 108 male subjects and the final score was compared in a linear fashion with a number of clinical parameters. For an RNA down-regulated in the original training classification data set (i.e., down-regulated between 25 years to 65 years) the ULSAM subject with the highest expression was assigned a score of 1 and the subject with the lowest expression 108. For genes up-regulated in the original age classification model, the opposite strategy was used. Thus, both feature selection (genes) and direction of regulation were taken from the original model. The median sum of these rank scores (reflecting the 150 probe-sets) was calculated and that represented the healthy ageing gene score for each individual in the ULSAM cohort. Median rank ensured each gene provided equal weighting and regression analysis was used to study the variation in gene score in these men, all of who had approximately the same chronological age.

The relationship between the gene score at age 70 years and a number of clinical features was carried out using multi-factor models. Model selection was executed using a forward selection approach, with *p* > 0.1 as stop criterion (backwards elimination yielded identical results). Clinical variables, previously reported [51], were added to the baseline model one at a time, and selected based on *p* value [96] (Additional file 2). Over the observation period mortality rate was 18 % (19 events) and the relationship between mortality and gene score was analyzed as a continuous variable. Both the Cox-regression and the logistic regression model were implemented in R. For the Cox model we used the latest ‘survival package’ whereas the logistic regression model was estimated using the glm (generalized linear model) function and ‘logit’ model, which models the log odds of the outcome as a linear combination of the predictor variables. For the Kaplan–Meier plots, gene score was divided into quartiles and the plot was produced using the plot-survfit function in the survival package. All three approaches yielded consistent results.

### Relationship between the healthy ageing gene score in blood and disease status using age- and gender-matched case–control analysis

Demonstration that the healthy ageing gene score was clearly demonstrable in neuro-muscular tissue suggested that it might also relate to cognitive health. Indeed, to provide additional support for the observations in human brain, we used the BrainEac.org gene-chip resource [52], which comprises ten post-mortem brain regions from 134 subjects representing 1,231 samples [GEO:GSE60862] (Additional file 1). For each brain region, and for a down-regulated gene in the original model, the subject with highest expression was assigned a score of 1 and the subject with the lowest expression was assigned a score of 134 (upper and lower score depends on total number of samples; Additional file 1). The median sum of the rank score was calculated for each anatomical brain region in the same manner as described above, with the ULSAM cohort. The healthy ageing gene score differed across the brain regions with chronological age, as determined by a Kruskal–Wallis test. A Kruskal–Wallis test was used as we were comparing unequal observations per age group (Additional file 1). Post hoc Mann–Whitney test with correction for multiple comparisons (Holm) was used to confirm regulation of the ageing signature genes in each region.

We used blood RNA profiles from subjects from the AddNeuroMed consortium, a large cross-European AD biomarker study and a follow-on Dementia Case Register (DCR) cohort in London. Patient selection, design, and clinical data have been reported previously [53, 97]. AD data sets have been deposited under accessions [GEO:GSE63060] and [GEO:GSE63061]. A summary of the cohort characteristics can be found in Table 1. Briefly, subjects were excluded from the study if they had neurological or psychiatric illness other than AD, unstable systematic illness or organ failure, or a geriatric depression rating scale score ≥ 4/5. AD was diagnosed using the National Institute of Neurological and Communicative Disease and Stroke and Alzheimer’s disease (NINCDS-ADRDA) and Diagnostic and Statistical Manual of Mental Disorders (DSM-IV) criteria for possible or probable AD. All MCI subjects reported problems with memory, corroborated by an informant, but had normal activities of daily living as specified in the Petersen’s criteria for amnestic MCI [41, 97]. All subjects underwent a structured interview and a battery of neuropsychological assessments, including the mini-mental state examination (MMSE). Control and MCI subjects were further assessed using the CERAD battery and detailed information on subject recruitment and assessments can be found in other published studies describing the AddNeuroMed consortium [54, 97]. RNA was obtained from whole venous blood and it was collected from the subjects who had fasted 2 hours prior to collection into a PAXgene™ Blood RNA tube (Becton & Dickenson, QIAGEN Inc., Valencia, CA, USA). The tubes were frozen at −20 °C overnight prior to long-term storage at −80 °C. RNA was extracted using PAXgene™ Blood RNA Kit (QIAGEN) according to the manufacturer’s instructions.

We used two independently produced gene-chip data sets from the AddNeuroMed/DCR consortia, one data-set produced in a UK gene-chip facility and another produced in the USA. Gene expression data was produced using Illumina Human HT-12 v.3 Expression BeadChips for the first case–control study (USA; cohort 1) and Illumina Human HT-12 v4 Expression BeadChips for the second case–control study (UK; cohort 2). cDNA was synthesized from 200 ng total RNA using the TotalPrep™ RNA Amplification Kit (Ambion), which was followed by amplification and biotinylation of cRNA and hybridization. The expression data were first transformed using variance stabilization and then quantile normalized using the LUMI package in R. The 150 probe-sets were mapped from the Affymetrix platform to the Illumina platform. For our primary analysis, control subjects were matched in a manner that created the largest possible group with the same chronological age and gender balance as the AD or MCI groups. Thus, our analysis was carried out on a subset of subjects deposited at the GEO, with each case–control group having a similar median chronological age as the ULSAM cohort. In total 297 samples were utilized (batch 1 Control = 67, MCI = 39, AD = 49; batch 2 Control = 72, MCI = 30, AD = 40). Retrospective inclusion of the entire cohort (n = 717) did not alter the outcome of our analysis.

We also utilized two additional large gene-chip clinical studies; one comparing blood RNA in type II diabetes with control [98] and the other from our laboratory comparing blood RNA in people with and without coronary artery disease [99]. For each case–control comparison the ranking metric was computed in the exact same manner as for the ULSAM subjects and AD patients (see above). A Wilcoxon rank sum test from the R stats package was used to test if the median gene score ranks between groups were significantly different or not. For data presentation, ranking scores were scaled to the total number of samples being ranked to ensure each data plot was on the same scale.

The bioinformatics tool Ingenuity Pathway Analysis (IPA) [100] was used to explore the biology of the age classifier genes. HUGO gene name identifiers were uploaded into IPA and queried against the verified IPA knowledge database. To establish the GO profile of the 150 genes, we generated a null distribution of GO enrichment *p* values by randomly sampling 10,000 lists of 150 probe-sets from the hgu133plus2 chip and testing each list for the GO term molecular function using the GOstats package in R. The entire population of probe-sets on the hgu133plus2 microarray was used as the background population for these tests. The resulting *p* values for each tested probe-set list were corrected using the method of Benjamini and Hochberg. The 150 healthy ageing genes were then tested for GO term molecular function and the *p* values Benjamini and Hochberg corrected. Positional gene enrichment analysis was used to identify whether the classification genes (or the classifier network genes) were significantly enriched within given chromosomal regions [55] as previously implemented [8].

## Additional files

Additional file 1:
**An Excel spreadsheet containing data related to our study with six tabs: 1) analysis of the top 150 genes from age prototype classifier in PUBMED; 2) the top 670 genes from the first stage of the project; 3) phenodata for the training data set and validation data sets in our study; 4) a list of prior markers in the literature for Alzheimer’s disease; 5) positional gene enrichment analysis; 6) sample information for the BrainEac study.** (XLSX 221 kb) Additional file 2:
**Table S1.** Clinical regression data from the ULSAM cohort. (DOCX 127 kb) Additional file 3:
**Figure S1.** A cumulative ranking metric of the healthy ageing metric was prognostic for mortality over a 20-year follow-up period. One-hundred and eight subjects provided a healthy tissue biopsy in 1992 that was suitable for RNA profiling and the fully annotated mortality data, covering 2009–2011, was retrieved from the Swedish national health registry. **a** The rank score for healthy ageing gene expression was calculated from the top 150 genes of the healthy ageing prototype classifier (n = 108, male subjects all ~70 years of age). Logistic regression analysis performed using the cumulative ranking metric of the top 150 genes from original prototype was prognostic for mortality. It showed that those subjects with the lowest median healthy ageing gene score had a much higher probability of death during the 20-year follow-up period (*p* = 0.0295). In contrast, members of the inflammatory response (GO:0006954) and mitochondrion (GO:0005739) gene ontology families - selected from ENSEMBL (BioMart) - showed no significant relationship with health during the 20-year follow-up period (*p* = 0.34 and *p* = 0.17). **b** The rank score for healthy ageing gene expression was calculated from the top 150 genes of the healthy ageing prototype classifier (n = 108, male subjects all ~70 years of age) and Kaplan–Meier plots were used to illustrate the temporal pattern of survival. Gene score was divided into quartiles and the plot was produced using the plot-survfit function in the R survival package. The plot allows us to compare overall survival rates between the four quartiles for gene score. The third and fourth quartiles differed from the first quartile (*p* < 0.04). (PDF 46 kb) Additional file 4:
**Figure S2.** Diabetes and vascular disease plots. The healthy aging signature activation was studied in blood samples from two independent large case–control studies of diabetes and vascular disease. Applying a Wilcoxon rank sum test, neither diabetes nor vascular disease was related to the healthy ageing gene score. This is consistent with our original hypothesis, and methods, that the healthy ageing gene score is not related to lifestyle factors and it is also consistent with the results observed in the ULSAM cohort (Fig. 3). **a** The diabetes data (94 controls versus 50 cases, group mean age = 66 years) originates from Tabassum et al*.* [98] (using Illumina Human HT.12.V4 arrays). **b** The vascular disease data (112 controls and 110 cases, group age = 53.3 years) originates from Sinnaeve et al*.* [99] (using Affymetrix HG-U133A arrays). (PDF 44 kb)

## Abbreviations

AD: Alzheimer’s disease
AUC: area under the curve
DCR: Dementia Case Register
fRMA: Frozen Robust Multi-array Analysis
GEO: Gene Expression Omnibus
GO: gene ontology
IPA: Ingenuity Pathway Analysis
kNN: *k*-nearest neighbor
LOOCV: leave-one-out cross-validation
MCI: mild cognitive impairment
NUSE: normalized unscaled standard error
RMA: Robust Multi-array Analysis
ROC: receiver operating characteristic
SNP: single nucleotide polymorphism
ULSAM: Uppsala Longitudinal Study of Adult Men
