# Supplementary material for: A novel multi-tissue RNA diagnostic of healthy ageing relates to cognitive health status
Source: Genome Biol. 2015 Sep 7;16(1):185. doi: 10.1186/s13059-015-0750-x (PMC4561473; doi:10.1186/s13059-015-0750-x)
Supplement: Additional file 2: — Table S1. Clinical regression data from the ULSAM cohort. (DOCX 127 kb) [file 13059_2015_750_MOESM2_ESM.docx]

**Table S1**

| Variable | Number of obs. | Mean@70y | SD | R | R^2^ | P-value |
| --- | --- | --- | --- | --- | --- | --- |
| **Cystatin C calculated GFR (ml/min)** | 123 | 64 | 12 | 0.48 | 0.110 | 0.0006 |
| **BMI (kg/m2)** | 128 | 25.8 | 2.8 | -1.43 | 0.052 | 0.0172 |
| **s-Albumin (g/l)** | 126 | 59.9 | 32.1 | -0.12 | 0.045 | 0.0221 |
| **Weight (kg)** | 128 | 78.9 | 9.9 | -0.37 | 0.042 | 0.0338 |
| **OGTT p-gluc 60 min (mmol/l)** | 128 | 9.6 | 2.6 | -1.14 | 0.028 | 0.0834 |
| **s-Phosphate (mmol/l)** | 127 | 43.0 | 2.3 | 1.26 | 0.025 | 0.1036 |
| **OGTT p-insulin AUC** | 128 | 1.4 | 0.8 | -3.38 | 0.023 | 0.1195 |
| **OGTT p-gluc 120 min (mmol/l)** | 128 | 7.2 | 2.7 | -0.78 | 0.015 | 0.2164 |
| **Free fatty acids (mmol/l)** | 128 | 4,0 | 1,0 | 2.14 | 0.014 | 0.2270 |
| **OGTT p-gluc 30 min (mmol/l)** | 128 | 9.1 | 1.6 | -1.26 | 0.013 | 0.2400 |
| **Interleukin-6 (ng/l)** | 122 | 3.9 | 4.9 | 0.40 | 0.014 | 0.2432 |
| **HDL cholesterol (mmol/l)** | 125 | 0.5 | 0.2 | -8.25 | 0.015 | 0.2558 |
| **s-Cholesterol (mmol/l)** | 128 | 1.3 | 0.3 | 6.07 | 0.012 | 0.2577 |
| **Systolic blood pressure supine (mmHg)** | 128 | 145 | 19 | -0.10 | 0.010 | 0.2969 |
| **Leisure time physical activity** | 125 | 3* |  | 2.99 | 0.010 | 0.3221 |
| **u-Albumin excretion rate (µg/min)** | 122 | 11.8 | 37.1 | -0.05 | 0.009 | 0.3393 |
| **s-Triglycerides (mmol/l)** | 128 | 6.0 | 1.1 | 1.43 | 0.008 | 0.3648 |
| **s-Insulin (pmol/l)** | 124 | 45.3 | 20.7 | -0.08 | 0.008 | 0.3673 |
| **OGTT p-gluc 0 min (mmol/l)** | 128 | 5.5 | 1.0 | 1.20 | 0.004 | 0.5099 |
| **Diastolic blood pressure supine (mmHg)** | 128 | 84 | 9 | -0.13 | 0.004 | 0.5143 |
| **Pulse rate (beats/min)** | 128 | 65 | 9 | -0.13 | 0.004 | 0.5149 |
| **Mini Mental State examination** | 121 | 28* |  | 0.07 | 0.002 | 0.6276 |
| **s-Creatinine (mol/l)** | 127 | 340 | 64 | 0.01 | 0.002 | 0.6474 |
| **s-Uric acid (mol/l)** | 125 | 1.0 | 0.3 | 2.04 | 0.001 | 0.7157 |
| **C-reactive protein (mg/l)** | 124 | 2.6 | 2.7 | 0.16 | 0.001 | 0.7972 |
| **LDL cholesterol (mmol/l)** | 126 | 80.2 | 30.8 | 0.01 | 0.0005 | 0.8272 |

**Table S1:** **Univariate linear regression on baseline characteristics in ULSAM at 70 years of age versus healthy age gene score.** Number of obs. denotes the number of complete observations available for each variable. Mean and SD denote mean and standard deviation respectively, variables marked with * are categorical and hence reported using median. R denotes the regression-coefficient of the variable. R2 and P-value denote r-squared and p-value of the univariate analysis.

**Figure S1. A cumulative ranking metric of the healthy-age metric was prognostic for mortality over a 20 y follow-up period.** One-hundred and eight subjects provide a healthy tissue biopsy in 1992 that was suitable for RNA profiling and the fully annotated mortality-data, covering 2009-2011, was retrieved from the Swedish national health registry. **S1A)** The rank score for healthy ageing gene expression was calculated from the top 150 genes of the healthy age prototype classifier (n=108, male subjects all ~70 years of age). Logistic regression analysis performed using the cumulative ranking metric of top 150 genes from original prototype was prognostic for mortality. It showed that those subjects with the lowest median healthy-aging gene score had a much higher probability of death during the 20 y follow-up period (p=0.0295). In contrast members of the Inflammatory response (GO:0006954) and Mitochondrion (GO:0005739) gene ontology families were selected from ENSEMBL (BioMart) and showed no significant relationship with health during the 20 y follow-up period (p=0.34 and p=0.17). **S1B)** The rank score for healthy ageing gene expression was calculated from the top 150 genes of the healthy age prototype classifier (n=108, male subjects all ~70 years of age) and Kaplan-Meier plots were used to illustrate the temporal pattern of survival. Gene-score was divided into quartiles and the plot was produced using the ‘plot-survfit’ function in the R survival package. The plot allows us to compare overall survival rates between the four quartiles for gene-score. The 3^rd^ and 4^th^ quartiles differed from the 1^st^ quartile (p< 0.04).

**Figure S2. Diabetes and Vascular disease plots.** The healthy aging signature activation was studied in blood samples from two independent large case-control studies of diabetes and vascular disease. Applying a Wilcoxon rank-sum test neither diabetes or vascular disease was related to the healthy ageing gene score. This is consistent with our original hypothesis that this gene score is not related to life-style factors and it is also consistent with the results observed in the ULSAM cohort (Figure 3). **S2A)** The diabetes data (94 controls versus 50 cases, group mean age=66y) originates from the Tabassum et al (J of Personalized Medicine *2014* using Illumina.Human.HT.12.V4 arrays). **S2B)** the vascular disease data (112 controls and 110 cases, group age=53.3yr) originates from Sinnaeve et al (PLoS One 2009 using Affymetrix HG-U133A arrays).
