# Supplementary figures and images for: A novel multi-tissue RNA diagnostic of healthy ageing relates to cognitive health status
Source: Genome Biol. 2015 Sep 7;16(1):185. doi: 10.1186/s13059-015-0750-x (PMC4561473; doi:10.1186/s13059-015-0750-x)

**A**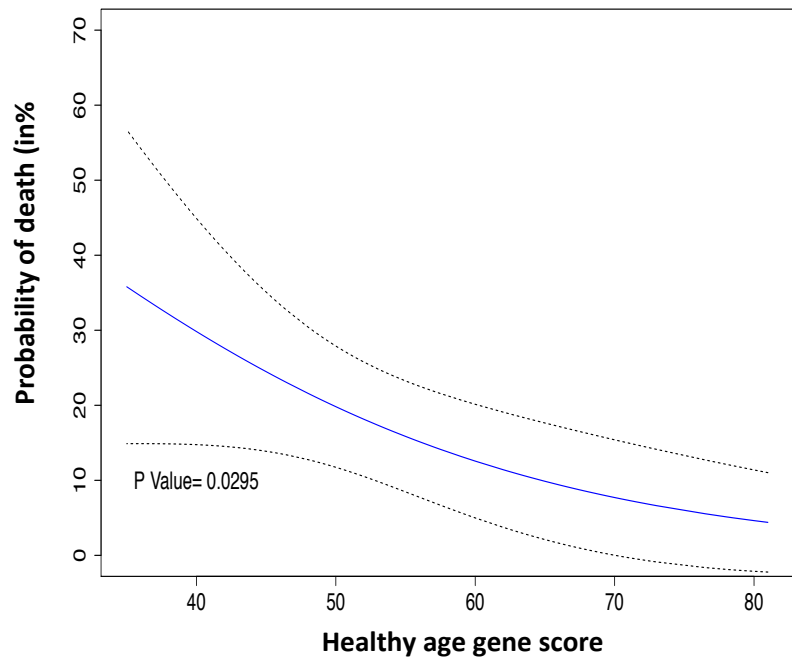**B**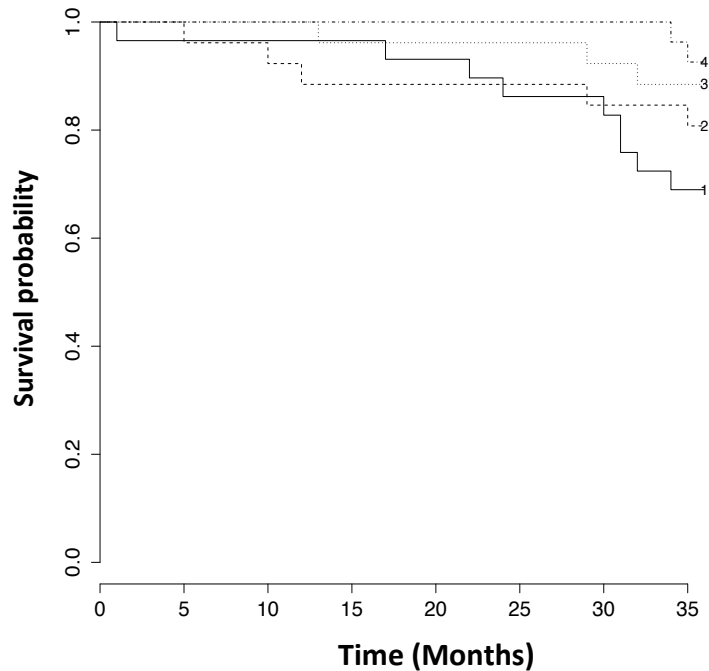

Supplement: Additional file 3: — Figure S1. A cumulative ranking metric of the healthy ageing metric was prognostic for mortality over a 20-year follow-up period. One-hundred and eight subjects provided a healthy tissue biopsy in 1992 that was suitable for RNA profiling and the fully annotated mortality data, covering 2009–2011, was retrieved from the Swedish national health registry. a The rank score for healthy ageing gene expression was calculated from the top 150 genes of the healthy ageing prototype classifier (n = 108, male subjects all ~70 years of age). Logistic regression analysis performed using the cumulative ranking metric of the top 150 genes from original prototype was prognostic for mortality. It showed that those subjects with the lowest median healthy ageing gene score had a much higher probability of death during the 20-year follow-up period (p = 0.0295). In contrast, members of the inflammatory response (GO:0006954) and mitochondrion (GO:0005739) gene ontology families - selected from ENSEMBL (BioMart) - showed no significant relationship with health during the 20-year follow-up period (p = 0.34 and p = 0.17). b The rank score for healthy ageing gene expression was calculated from the top 150 genes of the healthy ageing prototype classifier (n = 108, male subjects all ~70 years of age) and Kaplan–Meier plots were used to illustrate the temporal pattern of survival. Gene score was divided into quartiles and the plot was produced using the plot-survfit function in the R survival package. The plot allows us to compare overall survival rates between the four quartiles for gene score. The third and fourth quartiles differed from the first quartile (p < 0.04). (PDF 46 kb) [file 13059_2015_750_MOESM3_ESM.pdf]

**A**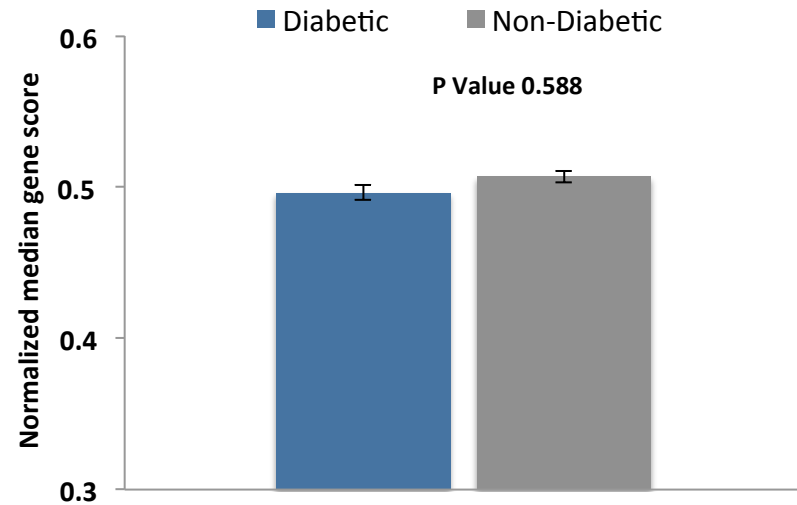**B**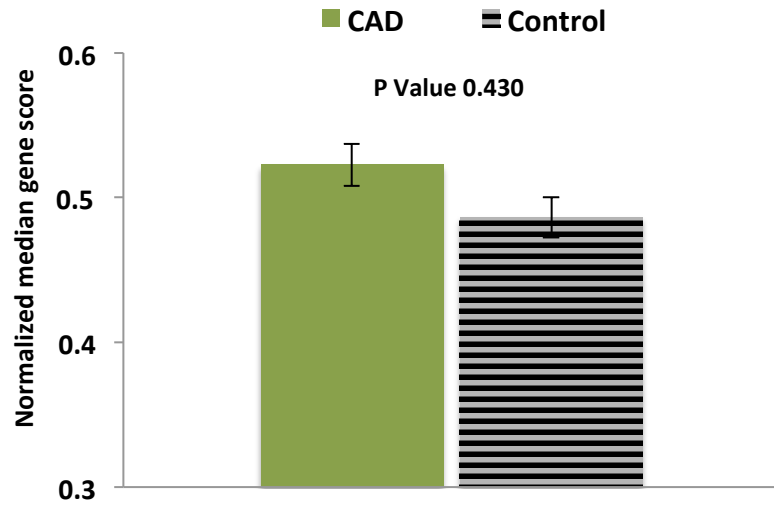

Supplement: Additional file 4: — Figure S2. Diabetes and vascular disease plots. The healthy aging signature activation was studied in blood samples from two independent large case–control studies of diabetes and vascular disease. Applying a Wilcoxon rank sum test, neither diabetes nor vascular disease was related to the healthy ageing gene score. This is consistent with our original hypothesis, and methods, that the healthy ageing gene score is not related to lifestyle factors and it is also consistent with the results observed in the ULSAM cohort (Fig. 3). a The diabetes data (94 controls versus 50 cases, group mean age = 66 years) originates from Tabassum et al. [98] (using Illumina Human HT.12.V4 arrays). b The vascular disease data (112 controls and 110 cases, group age = 53.3 years) originates from Sinnaeve et al. [99] (using Affymetrix HG-U133A arrays). (PDF 44 kb) [file 13059_2015_750_MOESM4_ESM.pdf]
